# Neutrophil elastase-cleaved corticosteroid-binding globulin is absent in human plasma

**DOI:** 10.1530/JOE-18-0479

**Published:** 2018-09-28

**Authors:** Lesley A Hill, Dimitra A Vassiliadi, Ioanna Dimopoulou, Anna J Anderson, Luke D Boyle, Alixe H M Kilgour, Roland H Stimson, Yoan Machado, Christopher M Overall, Brian R Walker, John G Lewis, Geoffrey L Hammond

**Affiliations:** 1 Departments of Cellular and Physiological Sciences and Obstetrics and Gynaecology, The University of British Columbia, Vancouver, British Columbia, Canada; 2 Endocrine Unit, Second Department of Internal Medicine-Research Institute and Diabetes Center, Attiko University Hospital, Athens, Greece; 3 BHF Centre for Cardiovascular Science, Queen’s Medical Research Institute, University of Edinburgh, Edinburgh, United Kingdom; 4 Department of Biochemistry and Molecular Biology, The University of British Columbia, Vancouver, British Columbia, Canada; 5 Institute of Genetic Medicine, Newcastle University, Newcastle upon Tyne, United Kingdom; 6 Canterbury Health Laboratories, Christchurch, New Zealand

**Keywords:** cortisol, monoclonal antibody, ELISA, steroid binding

## Abstract

Corticosteroid-binding globulin (CBG) transports glucocorticoids in blood and is a serine protease inhibitor family member. Human CBG has a reactive center loop (RCL) which, when cleaved by neutrophil elastase (NE), disrupts its steroid-binding activity. Measurements of CBG levels are typically based on steroid-binding capacity or immunoassays. Discrepancies in ELISAs using monoclonal antibodies that discriminate between intact vs RCL-cleaved CBG have been interpreted as evidence that CBG with a cleaved RCL and low affinity for cortisol exists in the circulation. We examined the biochemical properties of plasma CBG in samples with discordant ELISA measurements and sought to identify RCL-cleaved CBG in human blood samples. Plasma CBG-binding capacity and ELISA values were consistent in arterial and venous blood draining skeletal muscle, liver and brain, as well as from a tissue (adipose) expected to contain activated neutrophils in obese individuals. Moreover, RCL-cleaved CBG was undetectable in plasma from critically ill patients, irrespective of whether their ELISA measurements were concordant or discordant. We found no evidence of RCL-cleaved CBG in plasma using a heat-dependent polymerization assay, and CBG that resists immunoprecipitation with a monoclonal antibody designed to specifically recognize an intact RCL, bound steroids with a high affinity. In addition, mass spectrometry confirmed the absence of NE-cleaved CBG in plasma in which ELISA values were highly discordant. Human CBG with a NE-cleaved RCL and low affinity for steroids is absent in blood samples, and CBG ELISA discrepancies likely reflect structural differences that alter epitopes recognized by specific monoclonal antibodies.

## Introduction

Corticosteroid-binding globulin (CBG) transports glucocorticoids and progesterone in human blood and regulates their access to target tissues (Hammond 2016*a*). Human CBG is also known as SERPINA6 because it shares structural similarity with clade A serine proteinase inhibitor (SERPINA) family members (Hammond *et al.* 1987). Many SERPINAs inhibit proteases released during infections and inflammation by ensnaring them after proteolysis of an exposed reactive center loop region (RCL) that is characteristic of the SERPIN structure (Gettins & Olson 2016). While CBG is not known to inhibit proteases, its RCL is cleaved by neutrophil elastase (NE) (Hammond *et al.* 1990), chymotrypsin (Lewis & Elder 2014) and *Pseudomonas aeruginosa* elastase (Simard *et al.* 2014), causing a conformational change that disrupts CBG steroid-binding activity (Simard *et al.* 2015). Proteolysis of CBG appears to occur during the onset of inflammation (Hill *et al.* 2016) and is thought to increase the amount of ‘free’ or non-protein-bound glucocorticoids locally, at sites of inflammation (Hammond 2016*b*).

Plasma CBG levels were first determined using a variety of radio-ligand saturation assays that can also be used to examine its steroid-binding properties (Hammond & Lahteenmaki 1983). The first immunoassays for human CBG measurements relied on the use of polyclonal antibodies and radiolabeled CBG (Robinson *et al.* 1985). Plasma CBG concentrations measured by radioimmunoassay (RIAs) correlate well with measurements of its cortisol-binding capacity, except in samples containing CBG variants with abnormal steroid-binding properties (Robinson & Hammond 1985, Smith *et al.* 1992, Emptoz-Bonneton *et al.* 2000, Perogamvros *et al.* 2010). More recently, ELISAs have been introduced that rely on the use of polyclonal antibodies as the immobilization reagent and monoclonal antibodies that recognize specific epitopes on the surface of CBG for its detection (Lewis *et al.* 2003, Lewis & Elder 2011). One of these ELISAs employs a monoclonal antibody (12G2) that detects an epitope that is unperturbed by structural changes caused by RCL proteolysis (Lewis *et al.* 2003), and comparisons between plasma CBG values obtained using this ELISA and a cortisol-binding capacity assay have been used to identify CBG variants with abnormal steroid-binding activity (Hill *et al.* 2012, Simard *et al.* 2015). Another ELISA has been developed based on the use of a detection monoclonal antibody (9G12) raised against a synthetic polypeptide that spans the RCL, and its epitope is lost when the RCL is cleaved by NE (Lewis & Elder 2011). When plasma CBG levels measured in these two different ELISAs are compared in healthy individuals (Lewis & Elder 2011, 2013) and patient groups (Nenke *et al.* 2015, 2016*a*,*b*,*c*, 2017*a*), discrepancies in values have been interpreted as evidence for the presence of CBG with a NE-cleaved RCL and low affinity for cortisol, but direct evidence for this is lacking.

We have therefore examined discrepancies in these ELISA measurements using several biochemical approaches and sought to identify RCL-cleaved forms of human CBG with low affinity for steroids in plasma from patients with inflammation and abnormally low CBG levels.

## Materials and methods

### Plasma samples

A subset of 20 plasma samples from the CROATIA-Korcula cohort described in a previously published GWAS (Bolton *et al.* 2014) were selected to represent the discordancy observed between CBG-binding assay and ELISA values (10 samples were discordant in the 9G12 ELISA) and subsequently reanalyzed. The demographics for the subset of CROATIA-Korcula samples utilized in this study are as follows: concordant (age 58 ± 12 years, seven males and three females) and discordant (age 52 ± 15 years, two males and eight females), respectively.

Arterial and venous (from veins draining skeletal muscle, adipose, liver and brain) plasma samples from healthy lean individuals or obese patients were obtained from two published studies (for internal jugular Kilgour *et al.* 2015 and hepatic vein samples Stimson *et al.* 2011, respectively) and one unpublished study (for samples from veins draining forearm skeletal muscle and abdominal subcutaneous adipose tissue, using previously published techniques Hughes *et al.* 2012). Samples were obtained during steady state D_4_-cortisol ± D_2_-cortisone tracer infusions and before any interventions. Blood flow was measured in indocyanine green infusion (liver), Xenon washout (adipose), venous occlusion plethysmography (skeletal muscle) and magnetic resonance angiography (internal jugular). Net uptake/release across tissues was calculated as arterio-venous difference in concentration multiplied by blood flow. Inclusion/exclusion criteria were as previously published (Stimson *et al.* 2011, Kilgour *et al.* 2015), and subject characteristics are in Supplementary Table 1 (see section on supplementary data given at the end of this article). Ethical committee approval and written informed consent were obtained.

In addition, 146 plasma samples were obtained for analysis from patients admitted to the medical-surgical adult Intensive Care Unit (ICU) of Attikon University Hospital. Severe sepsis and septic shock were defined according to the SCCM/ESICM/ACCP/ATS/SIS international sepsis definitions (Levy *et al.* 2003). Exclusion criteria were age less than 18 years; mechanical ventilation for more than 48 h before ICU admission; no intubation and mechanical ventilation during ICU stay; do-not-resuscitate clinical conditions; brain-death upon ICU entry; prior or current glucocorticoid use and HIV infection. ICU samples analyzed in the current study were collected as part of a larger protocol that was approved by the hospital’s ethics committee, and informed consent was obtained from patients’ relatives.

### Measurements of CBG

Measurements of the cortisol-binding capacity of human CBG and its steroid-binding affinity by Scatchard analysis were performed using [^3^H]-cortisol or [^3^H]-corticosterone (PerkinElmer Life Sciences), as described (Hammond & Lahteenmaki 1983).

The 12G2 and 9G12 ELISAs of CBG in human blood samples have been described (Lewis *et al.* 2003, Lewis & Elder 2011). Blood samples or purified CBG were diluted appropriately, and plasma with a known CBG concentration, based on cortisol-binding capacity, was serially diluted for ELISA standards.

Included within these assays are two reference plasma samples, in which the CBG-binding capacity has been determined. One of the samples is consistently discordant in the 9G12 ELISA, whereas the other is consistently concordant between all assays.

### Purification of plasma CBG

Plasma samples treated with DCC to remove endogenous steroids were applied to an 11β-hydroxy-andros-4-en-3-oxo-17β-carboxylic acid Sepharose affinity gel (Robinson *et al.* 1985). After washing the gel with 100 mM Tris–NaCl, pH 7.4, containing 1 mM EDTA, steroid-bound CBG was eluted with 1 μM cortisol, in the same buffer, for analysis.

### Immunoaffinity depletion of CBG with an intact RCL epitope

The reference plasma sample in which CBG measurement in the 9G12 ELISA is highly discordant with 12G2 ELISA values was incubated with Protein-A affinity-purified 9G12 Mab (that recognizes only CBG with an intact RCL) overnight at 4°C. The mixture was then incubated with purified rabbit anti-mouse IgG antibodies (Sigma-Aldrich) coupled to NHS-activated Sepharose for 4 h at room temperature (GE Healthcare). The Sepharose immunoaffinity gel was sedimented by centrifugation and the supernatant was retained for analysis of CBG by ELISAs and steroid-binding capacity, as described earlier. In addition, the plasma sample was applied to a steroid-affinity gel prior to immunoprecipitation, and the semi-purified CBG was subjected to ELISAs to confirm that the discrepancy in ELISA values remained prior to immunoprecipitation with 9G12, as above.

### Biochemical analyses of CBG

A heat-dependent polymerization assay was used to identify RCL-cleaved CBG in plasma samples (Gardill *et al.* 2012). In brief, plasma was subjected to incremental increases in temperature (0.1°C/s to 70°C), prior to non-denaturing PAGE, and proteins in the electrophoresis gel were transferred to PVDF membranes using a Trans-Blot turbo transfer system (BioRad). The blots were blocked with 5% milk-PBST and incubated overnight at 4°C with a rabbit anti-human CBG antiserum (Robinson *et al.* 1985) diluted 1:4000 in 5% milk-PBST. Immunoreactive CBG was detected using horseradish peroxidase-labeled goat anti-rabbit IgG antibody (1:10,000; Sigma-Aldrich) for 1 h at room temperature, and ECL reagent using an ImageQuant LAS4000 (GE Healthcare). As a control in this assay, corresponding plasma samples were incubated with NE (Elastin Products Company) under conditions that specifically cleave the RCL of human CBG (Simard *et al.* 2014).

Denaturing SDS-PAGE (10% resolving gels) was performed on steroid-affinity gel purified CBG preparations (see above). Western blots were blocked and probed as described above. Rabbit anti-human CBG antibodies were affinity purified from antiserum (Robinson *et al.* 1985) using an *E. coli* expressed human CBG-GST fusion protein (Gardill *et al.* 2012) coupled to NHS-activated Sepharose (GE Healthcare).

### Mass spectrometry

Steroid affinity-purified CBG (6 µg) was digested (100:1 w/w) with trypsin (Promega) for 16 h in ammonium bicarbonate buffer, pH 8. As a positive control, purified CBG was incubated with human NE (10 min, 37°C) prior to trypsin digestion. Tryptic peptides were desalted using C18 stage tips (Supelco) and analyzed by liquid chromatography using an Easy nLC-1000 (Thermo Fisher Scientific) coupled to an Impact II QTOF tandem mass spectrometer equipped with a CaptiveSpray ion source (Bruker Daltonics). Desalted peptides (1 µg) were loaded onto an in-house packed column with 1.9 µm C18 ReproSil-Pur (Dr Maisch GmbH) and resolved using a linear gradient of 5–24% buffer B (100% acetonitrile) over 60 min. For peptide identification, a top 17 method was used, with the normalized fragmentation energy at 27%. Survey and fragment spectra were analyzed with Byonic v 2.16.11 (Protein Metrics).

### Statistical analyses

Arterio-venous data were analyzed by Wilcoxon signed-rank test and by Mann–Whitney *U* test. ICU patient data were analyzed using ANOVA, followed by Dunnett’s *post hoc* tests to examine significant main effects. Data are expressed as mean ± s.d. and differences were considered significant at *P* ≤ 0.05. Correlations were analyzed using linear regression and the corresponding *r* ^2^-value and best-fit linear regression line are provided in the results and figures, respectively.

## Results

### Comparisons of ELISA and cortisol-binding capacity measurements of CBG

**Figure 1:**
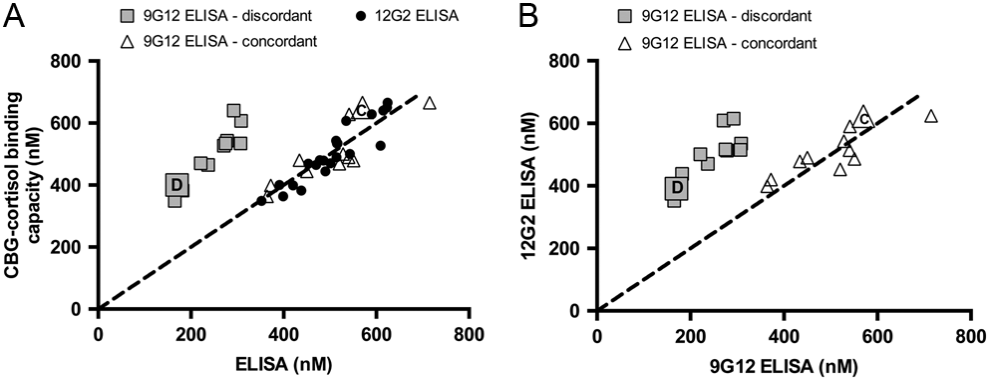
Despite differences in ELISA values in a subset of samples, CBG-cortisol binding capacities are normal. Comparisons of plasma CBG levels measured using two different ELISAs and a [^3^H]-cortisol-binding-capacity assay in 20 healthy individuals from the CROATIA-Korcula cohort. (A) In all samples, CBG values obtained by cortisol-binding capacity assay and 12G2 ELISA show a close correspondence (*circles*) with the dashed line indicating a perfect correspondence between values. In most samples, cortisol-binding capacity and 9G12 ELISA values closely align and are designated concordant (*triangles*). (A and B) In a subset of samples, 9G12 ELISA values are 50–60% lower than corresponding cortisol-binding capacity (A) or 12G2 ELISA values (B) and are designated discordant (*squares*). Included are two reference samples, in which the 9G12 ELISA and cortisol-binding capacity values are consistently either concordant (labeled C) or discordant (labeled D).

When ELISA and cortisol-binding capacity measurements of CBG in plasma samples from the CROATIA-Korcula cohort were analyzed, the 12G2 ELISA and cortisol-binding capacity measurements were always concordant, while 9G12 ELISA values in a subset of 10 individuals were ~50% lower than the corresponding cortisol-binding capacity values (Fig. 1A). Included within these assays are two reference plasma samples in which the 9G12 ELISA and cortisol-binding capacity values are consistently either concordant or discordant. These samples were also used as standards for comparisons of CBG ELISA and cortisol-binding capacity measurements in other studies (see below). When the two different ELISA values were compared with each other, a similar distribution of concordance and discordance was observed (Fig. 1B).

### Measurements of plasma CBG by ELISAs and cortisol-binding capacity are consistent in arterial and venous blood samples from the same individual

**Figure 2:**
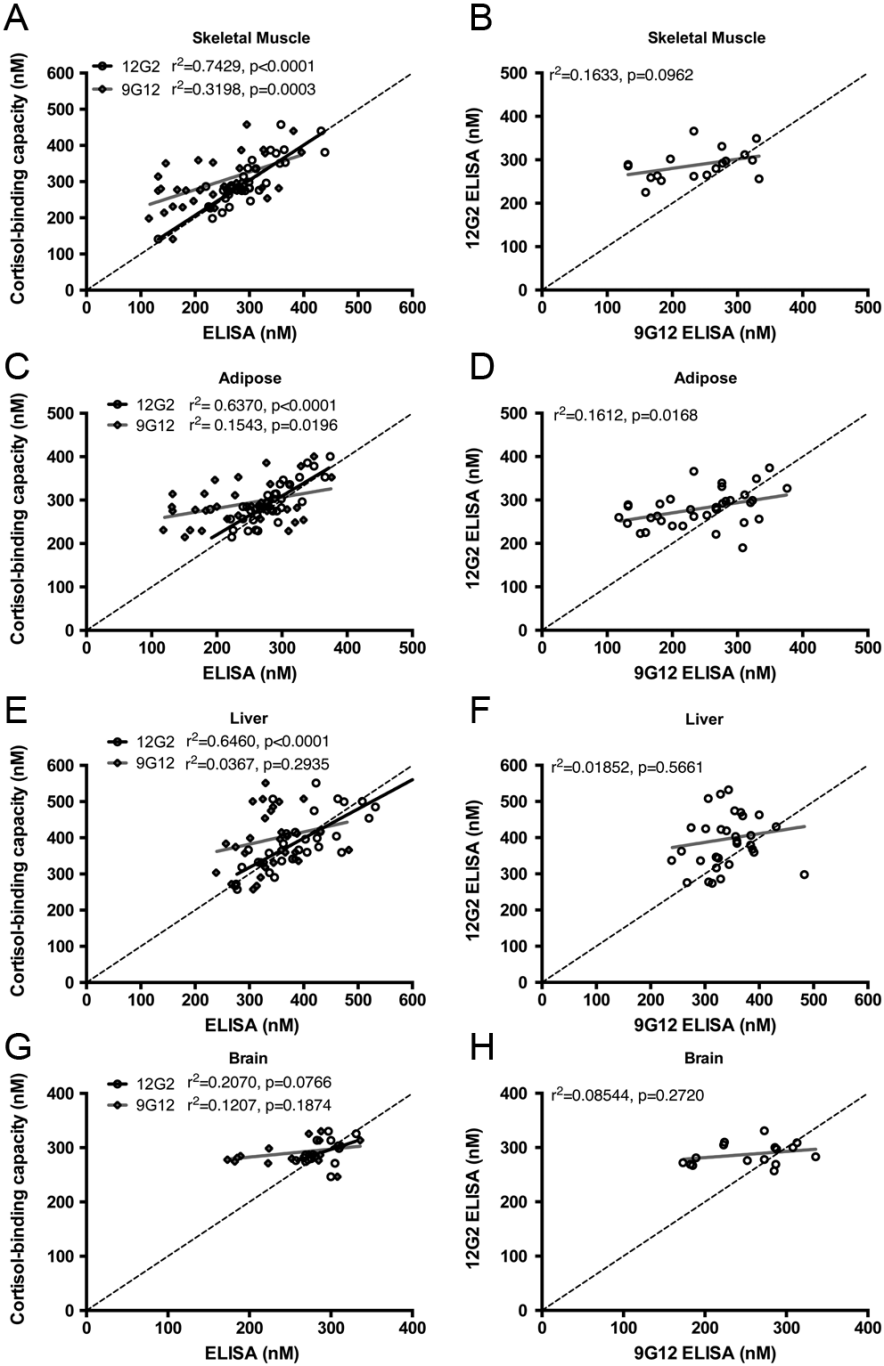
Plasma cortisol-binding capacity and 12G2 ELISA values show the strongest correlation. Blood samples were collected from a peripheral ‘arterialised’ vein from a heated hand (A, B, C, D, E, F, G and H), as well as from veins draining forearm skeletal muscle (A and B; *n* = 18), abdominal subcutaneous adipose (C and D; *n* = 18), liver (hepatic vein; E and F; *n* = 17) and brain (internal jugular vein; G and H; *n* = 8) veins and assayed for CBG levels. Data were analyzed using linear regression and the *r* ^2^ value and *P*-value are provided with each graph. (A, C, E and G) Within each group of combined arterial and venous samples from each cohort, CBG-cortisol binding capacity measurements were correlated more strongly with 12G2 ELISA values than with 9G12 ELISA values. (B, D, F and H) The correlation between the 12G2 and 9G12 ELISA values was also less strong than the correlation between 12G2 and cortisol-binding capacity measurements.

To determine if there were any variations in ELISA or cortisol-binding capacity measurements of CBG across individual tissues, arterial plasma samples and venous plasma samples from veins draining forearm skeletal muscle, subcutaneous adipose, liver and brain were collected. Of particular interest was adipose tissue, as differences in the levels of CBG across the tissue bed were anticipated in obese individuals, due to the presence of activated neutrophils. Within each group of samples, comparisons of arterial and venous CBG values obtained using the 12G2 ELISA with the corresponding cortisol-binding capacity values showed close correlations and concordance in skeletal muscle (Fig. 2A), adipose (Fig. 2C), liver (Fig. 2E) and brain (Fig. 2G). By contrast, the 9G12 ELISA values correlated less well with cortisol-binding capacity and 12G2 ELISA values in skeletal muscle (Fig. 2A and B), adipose (Fig. 2C and D), liver (Fig. 2E and F) and brain (Fig. 2G and H). The weak correlation between 9G12 ELISA values in comparison to cortisol-binding capacity and 12G2 ELISA values can be explained by ~25–50% lower 9G12 ELISA values in a subset of samples.

**Figure 3:**
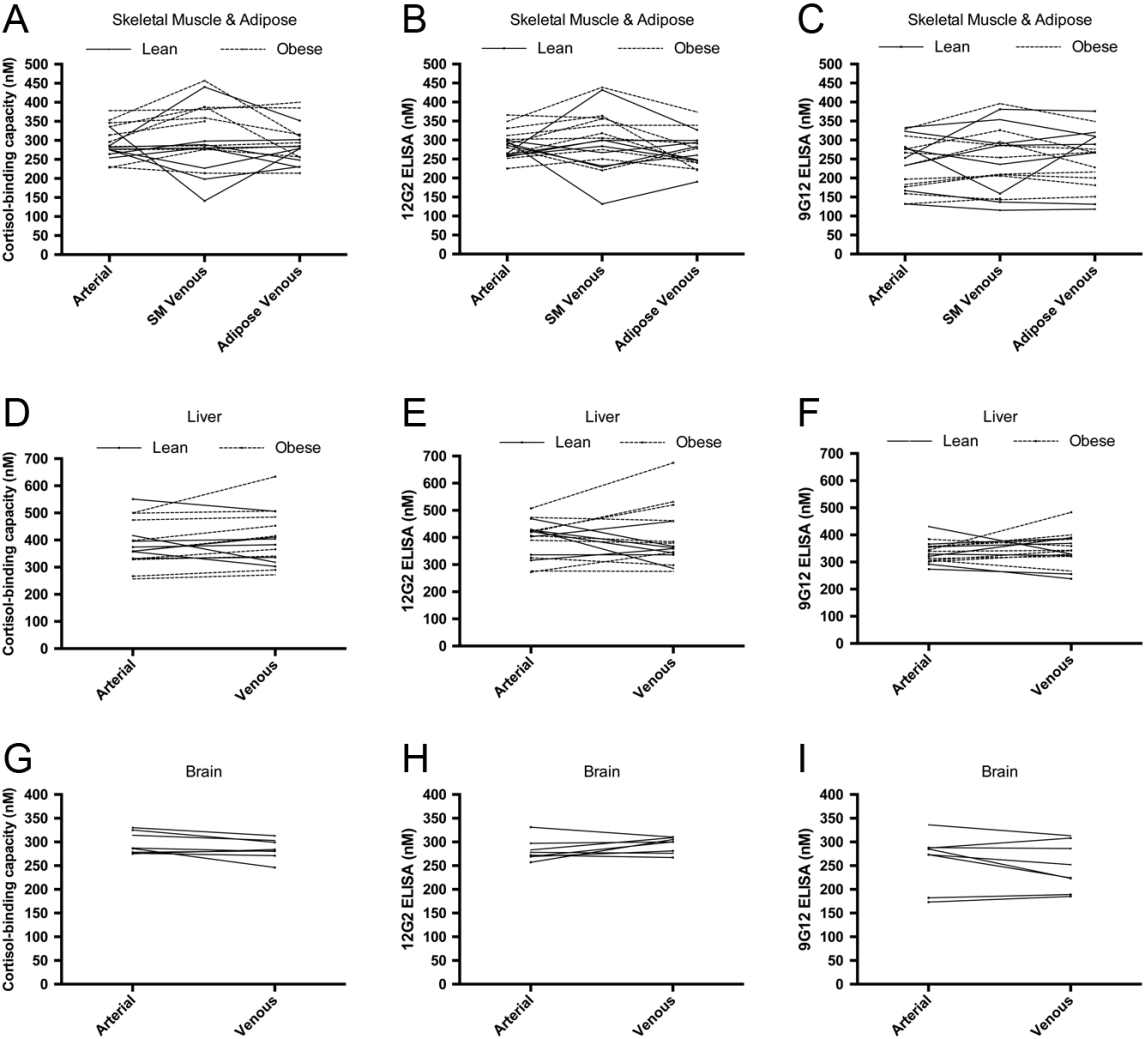
Plasma CBG measurements are consistent across different sites of blood sampling. Blood samples were collected from a peripheral ‘arterialised’ vein from a heated hand (A, B, C, D, E, F, G, H and I), as well as from veins draining forearm skeletal muscle (A, B and C; *n* = 18), abdominal subcutaneous adipose adipose (A, B and C; *n* = 17), liver (hepatic vein; D, E and F; *n* = 16) and brain (internal jugular vein; G, H and I; *n* = 8) veins, and assayed for CBG levels. Plasma CBG levels measured by cortisol-binding capacity (A, D and G), 12G2 ELISA (B, E and H) and 9G12 ELISA (C, F and I) are consistent across the different sites of blood sampling in a given individual.

Considering the arterio-venous differences across tissues, there were no gradients in cortisol-binding capacity measurements (Fig. 3A, D and G), 12G2 ELISA (Fig. 3B, E and H) or 9G12 ELISA values (Fig. 3C, F and I). In addition, after correction for blood flow, the only significant net uptake or release of CBG from individual tissues was detected across liver, where obese men with type 2 diabetes had net release of CBG as measured by cortisol-binding capacity (Supplementary Table 1). Notably, plasma CBG levels remained consistent when comparing arterial samples to blood collected from an adipose vein in a subset of obese individuals (Fig. 3A, B and C), suggesting that RCL cleavage does not occur across this tissue bed.

### No evidence for RCL cleavage of CBG in plasma samples

In an attempt to identify CBG with a cleaved RCL in blood, we first used a cortisol-binding capacity assay to measure plasma CBG levels in ICU patients (Supplementary Table 2 for patient demographics) with a variety of acute infections and/or inflammation, in whom activated neutrophils capable of cleaving the RCL of CBG were expected because of the severity of their disease.

**Figure 4:**
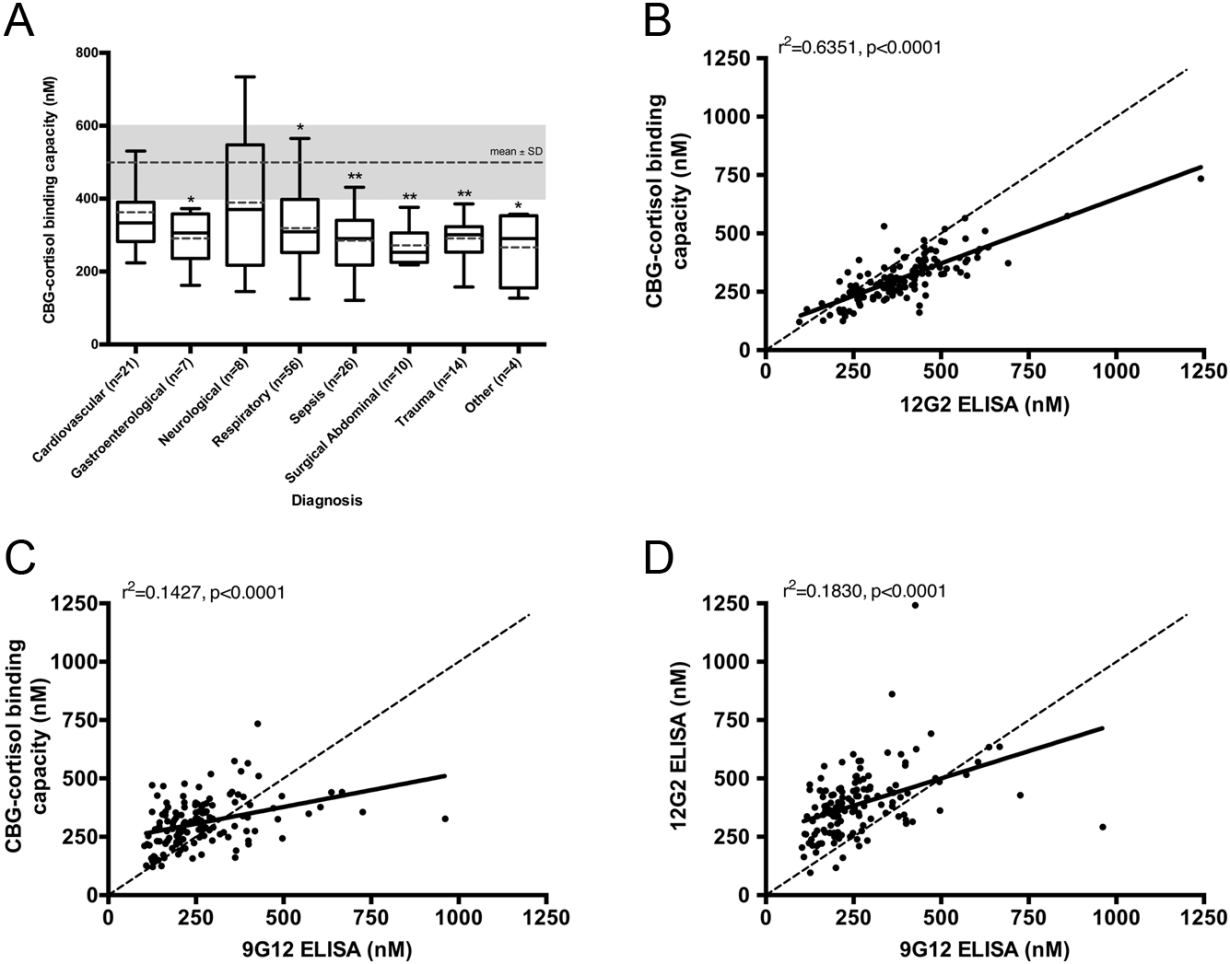
In ICU patient samples, decreased cortisol-binding capacity levels are observed, but a high correspondence with 12G2 ELISA values suggests RCL-cleaved CBG is not present. (A) Plasma CBG-cortisol binding capacity in various ICU patient populations. Box plots for each patient group display the maximum, third quartile, median, first quartile and minimum, with the mean included as a *dashed line*. CBG-cortisol binding capacity values were compared to the mean of a healthy control population (*mean* ± s.d.*, dashed line and shaded grey*), and significant differences were detected (main effect of diagnosis by ANOVA, *P* = 0.0039). Significantly lower CBG levels were found in patients with gastroenterological, respiratory and other diagnoses (*P < 0.05), as well as individuals with sepsis, surgical abdominal and trauma diagnoses (**P < 0.01). Data in A is presented as mean ± s.d. Dunnett’s *post hoc*: *P < 0.05, **P < 0.01. Plasma CBG levels were measured by cortisol-binding capacity (B), 12G2 (C) and 9G12 (D) ELISAs, and data were analyzed using linear regression, with *r* ^2^ values and *P*-values provided with each graph. (B) A strong correlation was found between cortisol-binding capacity and 12G2 ELISA values (*r* ^2^ = 0.6351). (C) Weak correlations were observed between 9G12 ELISA and cortisol-binding capacity (*r* ^2^ = 0.1427) or between the (D) 9G12 and 12G2 ELISA values (*r* ^2^ = 0.1830).

No significant differences in plasma CBG-cortisol binding capacity were found in the different patient populations (Fig. 4A). When compared to the average plasma CBG-cortisol-binding capacity of healthy individuals (*n* = 316) from the CROATIA-Korcula cohort (Bolton *et al.* 2014), significantly lower CBG-cortisol-binding capacity values were detected in the ICU patients (*P* = 0.0039), with significant differences found in sub-groups of patients with gastroenterological, respiratory and other diagnoses (*P* < 0.05), as well as in those with sepsis, surgical abdominal and trauma diagnoses (*P* < 0.01) (Fig. 4A).

When the 12G2 ELISA and cortisol-binding capacity assay values in these patients were compared (Fig. 4B), they correlated (*r* ^2^ = 0.635) and aligned well with each other. However, correlations between CBG levels determined by 9G12 ELISA, and CBG-cortisol-binding capacity (Fig. 4C) or 12G2 ELISA (Fig. 4D) were very weak (*r* ^2^ = 0.1830 and 0.1427, respectively), and this discordancy was unrelated to the severity of the patients’ clinical condition.

### Human CBG with a cleaved RCL is undetectable in ICU samples with discordant ELISA values

**Figure 5:**
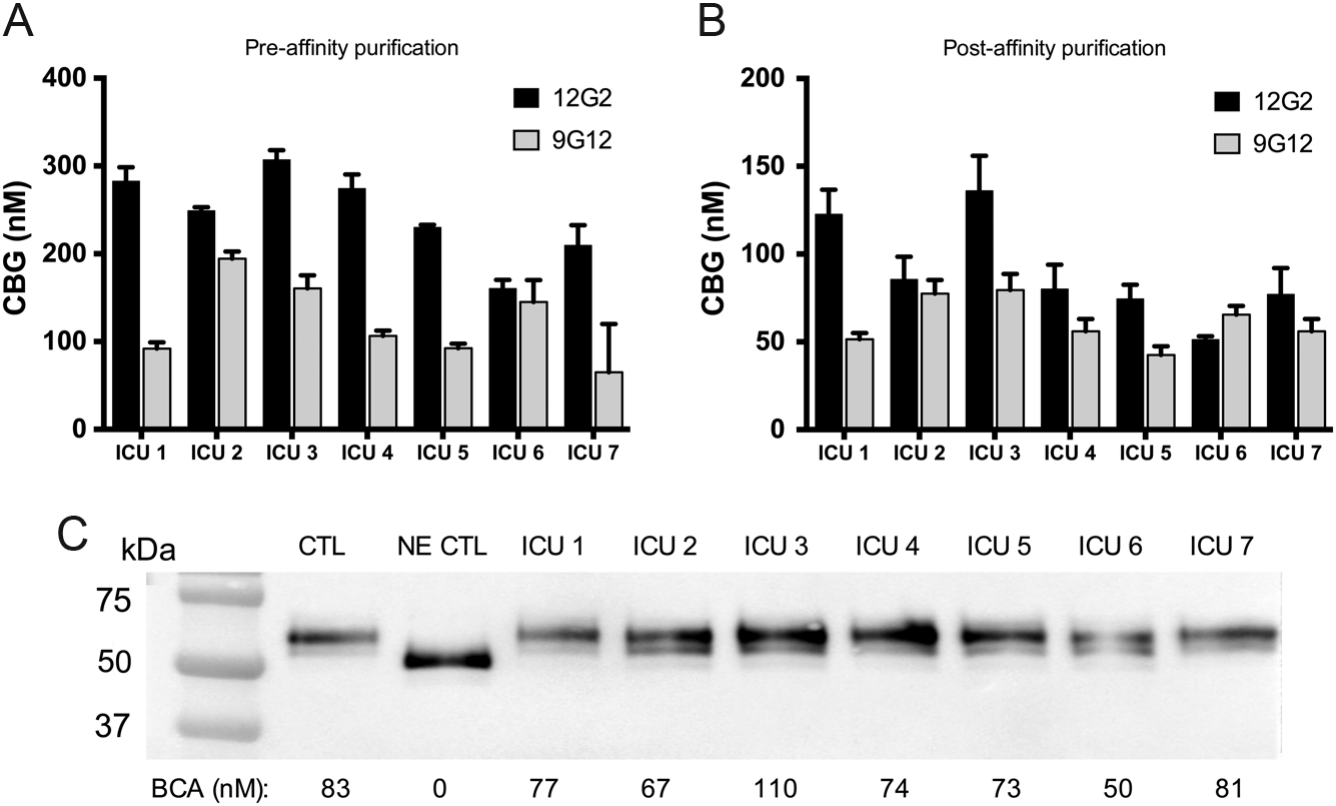
Following steroid-affinity purification, ICU samples remain discordant in the 9G12 ELISA, without evidence for CBG cleavage by western blotting. CBG from ICU patient plasma was purified using a steroid-affinity gel and assayed for CBG levels and protein integrity. The relationship between 12G2 and 9G12 ELISA values was consistent pre- (A) and post- (B) affinity purification; i.e. ICU samples that were concordant (ICU 2 and ICU 6) or discordant, respectively, remain so following purification. (C) Plasma CBG integrity was analyzed by western blotting. As controls, a sample from a healthy individual, untreated (CTL) or treated with NE (NE CTL), was included. As expected, following incubation with NE, the apparent molecular size of CBG shifted ~4 kDa downwards, and this shift was not observed in any of the ICU patient plasma samples. For each sample, cortisol-binding capacity assay values are provided for comparison; note the lack of CBG-cortisol binding capacity in the NE-treated sample.

Based on the premise that acutely ill patients with a discordancy in their plasma CBG levels by ELISAs reflects the presence of CBG with a cleaved RCL, we analyzed five ICU samples that exhibited a large discrepancy in 9G12 ELISA values and two ICU samples in which all CBG measurements were concordant. When the 12G2 and 9G12 ELISA values were compared in these samples pre- (Fig. 5A) and post- (Fig. 5B) steroid-affinity purification, a similar relationship was observed; two samples (ICU 2 and ICU 6) were concordant, while five ICU patients displayed a discordancy in the 9G12 ELISA values that ranged from 30 to 50% of the corresponding 12G2 ELISA values.

To optimize the resolution and accuracy of western blotting procedures, we affinity purified a rabbit anti-human CBG antibody (see Materials and methods) and used a steroid-affinity gel to isolate CBG from plasma samples. As a control, NE was used to cleave the RCL of CBG prior to steroid-affinity chromatography and western blot analysis. The resulting western blot shows that NE-cleaved CBG was retained and purified by the steroid-affinity gel despite its relatively low steroid-binding affinity, which was undetectable in the cortisol-binding capacity assay (Fig. 5C). As expected, the apparent molecular size of NE-cleaved CBG is also ~4 kDa smaller than the major immunoreactive forms of CBG in the ICU patient samples. Most importantly, the profiles of CBG in ICU patient plasma (e.g. ICU 2 and ICU 6) that show a concordance in their ELISA values, were identical to those observed in a normal control sample or in samples from patients that exhibit a discordance in their ELISA values, with no evidence of NE-cleaved CBG in any samples (Fig. 5C).

**Figure 6:**
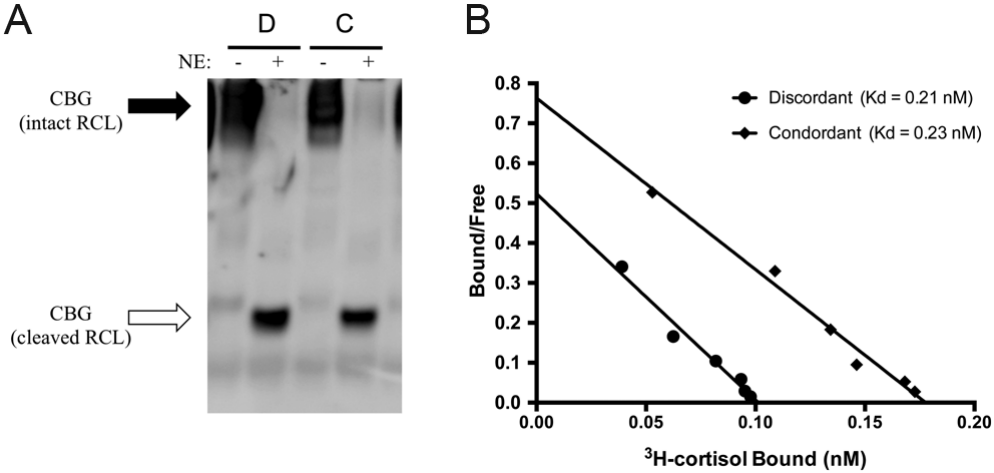
No evidence for RCL cleavage or low affinity CBG in a sample with a discordant CBG 9G12 ELISA value. (A) When analyzed in a heat-dependent polymerization assay, no differences were observed between a discordant (labeled D) and concordant (labeled C) sample. Under these conditions, CBG with an intact RCL polymerizes (solid arrow) while CBG with an RCL that is cleaved by NE does not (open arrow). This provides evidence for a lack of CBG cleavage in these samples, while NE cleavage clearly results in the expected changes in electrophoretic mobility in both samples. (B) No differences in the cortisol-binding affinity (Kd) of plasma CBG were observed in samples with concordant or discordant 9G12 ELISA values; Kd = 0.21 and 0.23 nM, respectively.

Samples in which 9G12 ELISA values were either concordant (C) or ~50% discordant (D), when compared with the corresponding cortisol-binding capacity or 12G2 ELISA values (Fig. 1), were also subjected to a heat-dependent polymerization assay that detects CBG with a cleaved RCL (Gardill *et al.* 2012). In this assay, CBG molecules with an intact RCL polymerize and run with a slow mobility during native PAGE, while the electrophoretic mobility of CBG with a cleaved RCL is much faster, and it migrates much further into the gel (Fig. 6A). This analysis demonstrated that while NE can effectively cleave CBG in both samples, there is no evidence of CBG proteolysis in the ELISA discordant sample. Furthermore, when subjected to Scatchard analysis (Fig. 6B), CBG in the concordant and discordant samples bound [^3^H]-cortisol with similar affinities (Kd=0.23 and 0.21 nM, respectively), and these values were similar to those measured in other samples using the same assay (Smith *et al.* 1992). In addition, the B_max_ value for sample C was almost twice as high as for sample D, which was in line with their respective cortisol-binding capacity and 12G2 ELISA values (Fig. 1C).

### Plasma CBG that is not recognized by the 9G12 Mab has a normal steroid-binding activity

**Figure 7:**
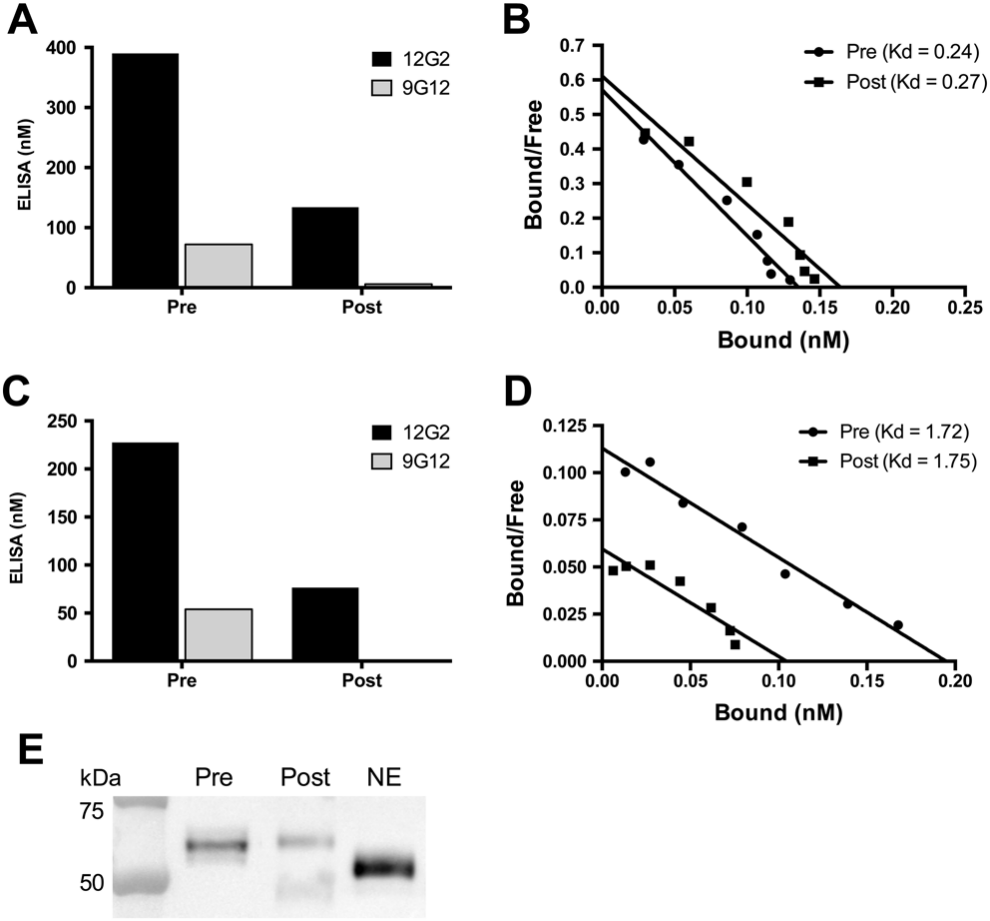
Plasma CBG that is not recognized in the 9G12 ELISA binds steroid with normal affinity and has a molecular size consistent with uncleaved CBG. Plasma samples were incubated with purified 9G12 to immunoprecipitate RCL-intact CBG using Sepharose-coupled rabbit anti-mouse IgG, and the supernatants were collected for analyses. (A) ELISA measurements were performed on a sample known to be discordant in the 9G12 ELISA, prior to immunoprecipitation (pre) and after 9G12-reactive CBG was immunoprecipitated (post). (B) The corresponding Scatchard plots of CBG in samples pre- and post- immunoprecipitation demonstrate a similar affinity for steroid (Kd = 0.24 and 0.27 nM, respectively). (C) This highly discordant sample was also subjected to steroid-affinity purification prior to 9G12 immunoprecipitation and the discordancy between the ELISA values was unchanged. Steroid-affinity purified CBG was detected in the 12G2 ELISA following immunoprecipitation with the 9G12 Mab but was not detectable using the 9G12 ELISA, as expected. (D) Scatchard analysis demonstrated that steroid-affinity purified CBG binds corticosterone with a similar affinity in the sample prior to (pre, Kd = 1.72 nM) and after (post, (Kd = 1.75 nM) 9G12 immunoprecipitation. (E) The integrity of CBG in the samples pre- and post- 9G12 immunoprecipitation was also assessed by western blotting and compared with NE-cleaved CBG that is characterized by an ~4 kDa reduction in molecular size. Note there is no evidence of immunoreactive CBG with a molecular size consistent with NE-cleavage, in either the pre- or post- 9G12 immunoprecipitation samples. In the post sample, there is a faint band at ~50 kDa, which is specifically recognized by the goat anti-rabbit IgG antiserum used for western blotting, and this represents a trace amount of rabbit anti-mouse IgG that leaches from the Sepharose immunoaffinity gel.

In addition, we sought to characterize the molecular and biochemical properties of CBG that is not recognized by the 9G12 Mab. To accomplish this, a reference plasma sample in which the 9G12 ELISA value is consistently ~50% discordant (Fig. 7A) was incubated with purified 9G12 Mab, and CBG-antibody complexes were immunoprecipitated with Sepharose coupled rabbit-anti mouse IgG. When CBG in the supernatant was analyzed in the ELISAs, it was readily detected using the 12G2 Mab, while 9G12 immunoreactive CBG levels were barely detectable (Fig. 7A). Therefore, if the 9G12 Mab only detects CBG with an intact RCL, the RCL of CBG in the supernatant after immunoprecipitation with the 9G12 Mab should be cleaved with a low steroid-binding affinity, but its affinity for corticosterone (Kd = 0.27 nM) was similar to that of plasma CBG (Kd = 0.24 nM) prior to immunoprecipitation (Fig. 7B).

In other experiments using the same ELISA discordant sample, CBG was first purified from plasma using a steroid-affinity gel, and measured in the 12G2 and 9G12 ELISAs to verify that the discordancy was preserved (Fig. 7C). The affinity-purified CBG was then immunoprecipitated using the 9G12 Mab and the remaining CBG in the supernatant was analyzed in the 12G2 and 9G12 ELISAs. In the supernatant, CBG was easily detected using the 12G2 Mab, while 9G12 immunoreactive CBG was undetectable (Fig. 7C). Similar to the results seen in plasma, Scatchard analysis demonstrated that the affinity of CBG for corticosterone in the supernatant (post immunoprecipitation, Kd = 1.75 nM) was similar to that of steroid-affinity gel purified CBG (pre immunoprecipitation, Kd = 1.72 nM) (Fig. 7D). A lack of cleaved CBG in the supernatant was confirmed using SDS-PAGE (Fig. 7E). The higher steroid-binding affinity of purified CBG in comparison to plasma is due to the absence of contaminating albumin, which alters the bound-to-free ratio in Scatchard plots.

### NE-cleaved CBG is not present in plasma with a discrepant 9G12 value

**Figure 8:**
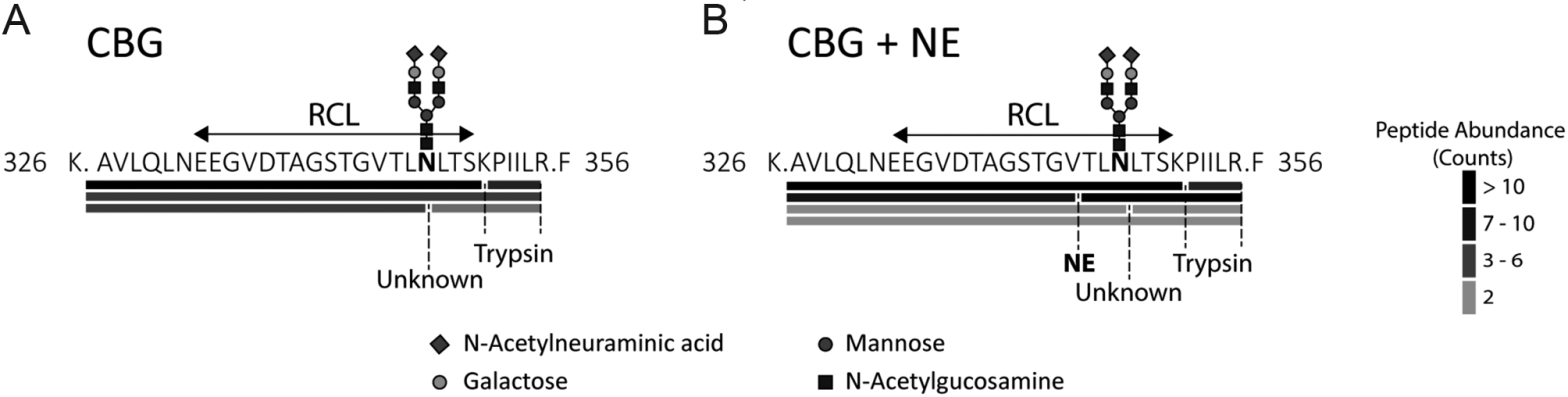
9G12 ELISA discrepant plasma CBG has an intact RCL as identified by high-resolution mass spectrometry. Affinity-purified plasma CBG was trypsin digested and the resulting peptides were identified by LC-MS and analyzed with Byonic v2.16.11. As a positive control for RCL cleavage, CBG was first incubated with NE (10 min). RCL spanning peptides identified by LC-MS in (A) plasma CBG and (B) NE-cleaved plasma CBG samples. Cleavage sites corresponding to NE, trypsin and an unknown protease are shown. Spectral counts were used as a surrogate of peptide abundance. At Asn347, the most common *N*-glycan identified was sialylated and biantennary, and this is shown for reference.

Finally, mass spectrometry and peptide analyses were used to determine if RCL-cleaved CBG is present in plasma with a highly discrepant 9G12 value. We obtained 93% sequence coverage of purified human plasma CBG. As a positive control, purified CBG was incubated with NE, and we identified the non-prime NE-cleaved semi-tryptic peptide (326K.AVLQLNEEGVDTAGSTGV.T345) (Fig. 8B; Supplementary Fig. 1C), as well as a prime side cleavage product (344V.TLNLTSKPIILR.F356) (Fig. 8B; Supplementary Fig. 1D). However, these peptides were not identified in untreated purified CBG (Fig. 8A; Supplementary Fig. 1A and B). In human plasma, the intact RCL peptide (326K.AVLQLNEEGVDTAGSTGVTLNLTSKPIILR.F356) spanning the NE cleavage site was the dominant peptide identified (Fig. 8A; Supplementary Fig. 1B).* N*-glycosylation at Asn347 was detected in tryptic peptides spanning the RCL, confirming occupancy of *N*-glycans at this site *in vivo*, with the major glycans observed being sialylated bi-antennary oligosaccharides. Low abundance peptides corresponding to a proteolytic processing event at Asn347 were identified, but this was not surprising given that low levels of spurious cleavage may occur during extensive CBG purification or analysis (Gardill *et al.* 2012). Hence, steady state levels of NE-cleaved CBG are not present in human plasma.

## Discussion

Proteolytically cleaved CBG has been observed in bronchoalveolar lavage fluid of patients with inflammatory lung disease, while CBG cleavage products were absent in serum from the same patients (Williams *et al.* 2009). Low serum levels of CBG have been demonstrated by CBG cortisol-binding capacity and RIA in patients with septic shock (Pugeat *et al.* 1989). Precipitous decreases in plasma CBG levels observed during acute infections, trauma or severe inflammation (Zouaghi 1984, Bernier *et al.* 1998) are thought to reflect NE cleavage of the CBG RCL and disruption of its steroid-binding affinity (Hammond *et al.* 1990). Although granulocytes from patients with severe acute inflammation also cleave and disrupt the steroid-binding activity of CBG, while those from healthy individuals do not (Hammond *et al.* 1990), the fate of CBG in the blood circulation of acutely ill patients after the proteolysis of its RCL remains enigmatic.

This is an important question, especially in light of numerous reports suggesting that RCL-cleaved CBG within the blood (Nenke *et al.* 2015, 2016*a*,*b*,*c*, 2017*a*,*b*) makes up to 30% of total plasma CBG, even in healthy individuals (Lewis & Elder 2011). However, reduced levels of RCL-cleaved CBG inferred from ELISA measurements in patients with abdominal obesity (Nenke *et al.* 2016*a*), alpha-1-antitrypsin deficiency (Nenke *et al.* 2016*b*) and active rheumatoid arthritis (Nenke *et al.* 2016*c*) are paradoxical and hard to reconcile because activated neutrophils in these patients would be expected to increase CBG cleavage. Evidence for RCL-cleaved CBG within the circulation in these studies has been based solely on differences between CBG measurements in two ELISAs that rely on different monoclonal antibodies for CBG detection (Lewis *et al.* 2003, Lewis & Elder 2011), and our biochemical analyses have now demonstrated that discrepancies in these two ELISAs do not reflect abnormalities in the steroid-binding affinity of human CBG or proteolysis of its RCL. Moreover, we have been unable to detect RCL-cleaved CBG in blood samples from acutely ill patients with very low plasma CBG levels or in blood samples in which there is a marked discrepancy in CBG levels measured using an ELISA that employs a monoclonal antibody designed to recognize CBG molecules with an intact RCL (Lewis & Elder 2011).

Assays of CBG based on functional measurements of its steroid-binding capacity or immunoreactivity both have inherent limitations. Steroid-binding capacity measurements rely on the saturation of a single steroid-binding site per CBG molecule, and the amount of bound ligand is therefore assumed to directly reflect the molar concentration of the protein. In our steroid-binding capacity assay, DCC is used to adsorb non-CBG bound radio-labeled ligand, and irreversible losses of CBG-bound ligands occur during this process and have to be accounted for (Hammond & Lahteenmaki 1983). This allows for the identification of CBG mutants with abnormally low steroid-binding affinity, and NE-cleaved CBG with an ~10-fold reduction in steroid-binding affinity is undetectable in this assay (Simard *et al.* 2015), as also demonstrated here (Fig. 5C). Thus, if the 9G12 ELISA detects only CBG with an intact RCL and a high affinity for cortisol, the 9G12 values should closely align with the cortisol-binding capacity values, and in many cases they do not.

By contrast, samples that show ~30–50% discordance between 9G12 ELISA and cortisol-binding capacity values, have corresponding 12G2 ELISA values that align closely with cortisol-binding capacity values. In the samples studied, we observed the strongest correlation between plasma CBG levels measured by cortisol-binding capacity and 12G2 ELISA, and this provided an indication that the discrepancy in ELISA values has nothing to do with an abnormality in steroid-binding activity, but rather hints at a variance in 9G12 monoclonal antibody recognition.

In most of the blood samples analyzed, the CBG measurements by the three different assays correlated and aligned well with each other, but highly discordant 9G12 ELISA values were observed in a subset of samples. This discordance in 9G12 ELISA values was unrelated to the site of blood collection or the health status of the individuals from whom the samples were taken. Moreover, this discordance in the ELISA or CBG-cortisol binding capacity values was consistently observed in samples collected at different anatomical locations, including skeletal muscle, adipose, liver and brain. Notably, CBG levels in obese individuals remained consistent across adipose tissue, in which activated neutrophils are expected to be present (Dam *et al.* 2016) and cause proteolysis of the CBG RCL.

Although it is clear that *in vitro* RCL-cleavage of CBG by NE disrupts high affinity cortisol binding (Pemberton *et al.* 1988, Hammond *et al.* 1990) and recognition by the 9G12 Mab (Lewis & Elder 2011), evidence that RCL-cleaved CBG is retained within blood is circumstantial. To address this, we utilized a heat-dependent polymerization assay that detects NE-mediated RCL cleavage of CBG (Gardill *et al.* 2012), but found no evidence of RCL-cleaved CBG in a sample in which the 9G12 ELISA value is highly discordant. Further evidence for a lack of RCL-cleaved CBG with a low steroid-binding affinity in this ELISA discrepant sample was obtained by Scatchard analyses.

We used a steroid-affinity gel to purify CBG from plasma samples taken from critically ill patients in whom CBG levels were lower than normal, as the most likely patient group in which cleaved CBG was to be expected. When CBG purified from these patients was analyzed, the concordance or discordance between their ELISA values remained, and there was no evidence of RCL-cleaved CBG with an increased electrophoretic mobility by western blotting. By examining the biochemical properties of CBG in samples with a discrepancy in ELISA values, we also demonstrate that CBG that is not recognized by the monoclonal antibody (9G12) directed against an intact RCL has a normal affinity for cortisol, and the same molecular size as CBG with an intact RCL. In other words, CBG that is undetected by the 9G12 ELISA has a high steroid-binding affinity and an intact RCL. The absence of NE-cleaved CBG in human plasma was further confirmed by high-resolution mass spectrometry.

While we present direct evidence that CBG with a cleaved RCL and a low affinity for cortisol is not present in human plasma samples, we still do not know what happens to CBG after its RCL is cleaved *in vivo*. Studies of the plasma clearance of native, RCL-cleaved and protease-complexed forms of SERPINA1, indicated that RCL-cleaved SERPINA1 is cleared from the circulation more rapidly than the intact protein, but not as rapidly as the SERPINA1–protease complex (Mast *et al.* 1991). Moreover, the mechanism responsible for the clearance of RCL-cleaved SERPINA1 appeared to be unrelated to that responsible for the clearance of SERPINA1-protease complexes (Mast *et al.* 1991), which is known to involve a multifunctional scavenging and signaling receptor, the low-density lipoprotein receptor-related protein 1 (Strickland *et al.* 2011). The plasma clearance of CBG and SERPINA1 may behave similarly after cleavage of their RCLs, but this remains to be determined.

Why CBG in blood samples from some individuals behave anomalously in the ELISAs we and others have used remains to be defined, but it is clear that the epitope recognized by the 9G12 monoclonal antibody includes the residues (STGVTLNL) between Ser341 and Leu348 of human CBG and spans the NE cleavage site after Val344 (Lewis & Elder 2011). The C-terminal portion of this epitope sequence also contains part of an *N*-glycosylation sequence (NL-T), which is utilized in approximately 85% of plasma CBG molecules (Sumer-Bayraktar *et al.* 2011). The *N*-glycan at this position is also heterogeneous with a relatively high degree of fucosylation (35%) and bi-, tri- or tetra-antennary oligosaccharide structures (Sumer-Bayraktar *et al.* 2011), and this variability has been reported to influence the protease recognition and/or cleavage of the RCL (Sumer-Bayraktar *et al.* 2016). This is particularly relevant because the synthetic polypeptide used to develop the 9G12 monoclonal antibody was not glycosylated, and recognition of this epitope is likely influenced by the presence or absence of different *N*-glycans attached to the RCL sequence. In support of this, a naturally occurring CBG T349A variant exhibits unexpectedly high values in the 9G12 ELISA when compared to the 12G2 ELISA (Simard *et al.* 2015). We attribute this apparent increase in the reactivity of CBG T349A in the 9G12 ELISA to the fact that Thr349 is located just outside of the 9G12 epitope and its substitution with alanine disrupts *N*-glycosylation at Asn347, making the epitope more accessible to the 9G12 monoclonal antibody. In contrast, the 12G2 monoclonal antibody was raised using CBG as the antigen and appears to recognize an epitope that includes Ala256, which was deduced from the observation that CBG A256T is not recognized in the 12G2 ELISA (Simard *et al.* 2015). The fact that this residue is located within a structurally constrained region on the surface of CBG may explain why there is an excellent correlation and alignment between 12G2 ELISA and steroid-binding capacity assay values.

Although *N*-glycosylation within the RCL might influence the ability of the 9G12 monoclonal antibody to recognize its epitope, the overall structure of the RCL is also thought to be dynamic and unstructured, and its positioning may be altered by occupancy of the steroid-binding site (Lin *et al.* 2009, 2010). Furthermore, we recently reported that quantitative and qualitative differences in *N*-glycosylation at other sites in the CBG molecule may influence its structure and steroid-binding properties, and predicted that this may influence CBG recognition by specific monoclonal antibodies (Simard *et al.* 2018). This might explain why measurements of CBG by RIAs, which use polyclonal antibodies that recognize a large number of epitopes, are closely aligned with cortisol-binding capacity measurements (Robinson & Hammond 1985).

Discrepancies in the ELISAs utilized have been associated with specific SNPs within the *SERPINA6* gene, and it appears that the recognition of CBG in the 9G12 ELISA is influenced by rs12589136, which is located upstream of the *SERPINA6* transcription start site close to a consensus estrogen response element (Bolton *et al.* 2014). Although it has been suggested that estrogen dependent increases in CBG production might account for differences in 9G12 monoclonal antibody recognition, for instance during pregnancy or oral contraceptive use (Nenke *et al.* 2017*b*), there are no obvious sex differences in the frequency of the discordancy in these ELISA measurements. It is therefore possible that linkage disequilibrium between rs12589136 and another polymorphism in another gene influences the structure of CBG, for instance as the result of a post-translational modification, in ways that alter 9G12 monoclonal antibody recognition of its epitope in the CBG RCL.

In summary, our studies, utilizing a variety of complementary, highly sensitive and orthogonal approaches, including high-resolution mass spectrometry, indicate that human CBG with a NE-cleaved RCL is undetectable in blood samples, even from acutely ill patients. Interestingly, we have demonstrated that during acute inflammation in rats, CBG with a cleaved RCL and undetectable steroid binding is retained in the blood circulation (Hill *et al.* 2016), and this is quite different to what we see in critically ill humans. It is possible that the structural changes in CBG after RCL cleavage during acute inflammation in humans and rats are different, or that there are species differences in the clearance mechanisms for RCL-cleaved CBG. The question of the fate of human CBG after RCL cleavage by NE therefore remains to be resolved.

## Supplementary Material

Supporting Figure 1

Supporting Table 1

Supporting Table 2

## Declaration of interest

The authors declare that there is no conflict of interest that could be perceived as prejudicing the impartiality of the research reported.

## Funding

This work was supported by the Canadian Institutes of Health Research (Operating Grant MOP-111102), the Wellcome Trust and the British Heart Foundation. G L H is recipient of the Tier 1 Canada Research Chair in Reproductive Health.

## Author contribution statement

A J A, A H M K, R H S and B R W designed and conducted the clinical studies. G L H and L A H designed the study. L A H, L D B and Y M analyzed the data, and L A H prepared the manuscript with input from G L H, D A V, I D, C M O, B R W, J G L. All authors have approved the research and contents of the manuscript.
