# Supplementary figures and images for: Neutrophil elastase-cleaved corticosteroid-binding globulin is absent in human plasma
Source: J Endocrinol. 2018 Sep 28;240(1):27–39. doi: 10.1530/JOE-18-0479 (PMC6347282; doi:10.1530/JOE-18-0479)

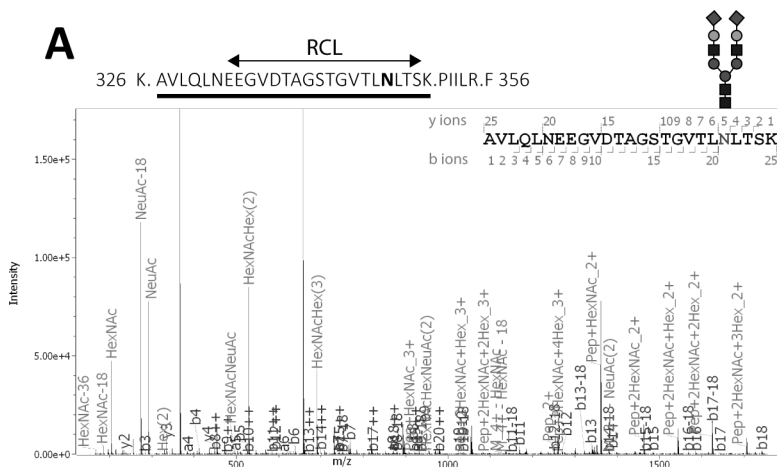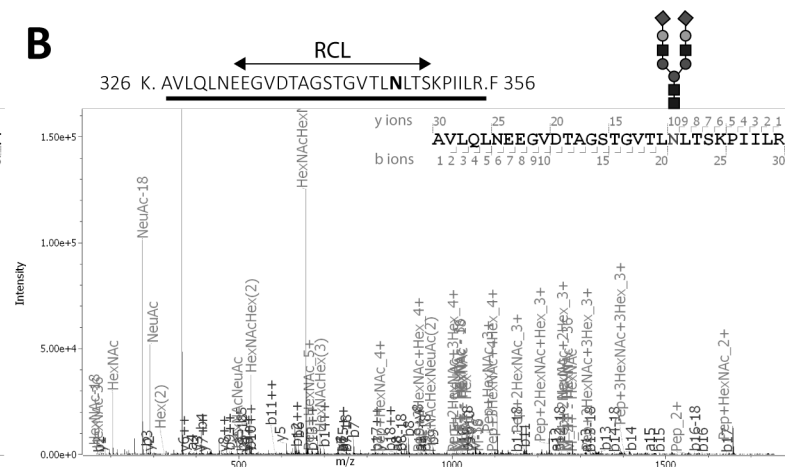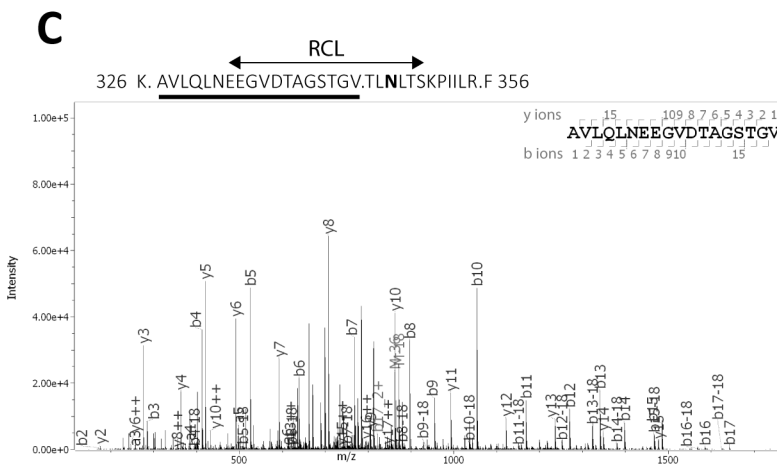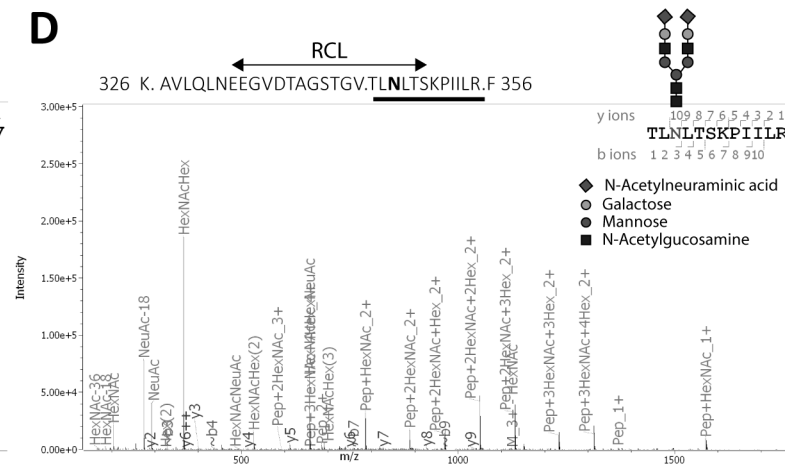

Supplement: Supporting Figure 1 [file JOE-18-0479-s001.pdf]
