# Supplementary material for: Neutrophil elastase-cleaved corticosteroid-binding globulin is absent in human plasma
Source: J Endocrinol. 2018 Sep 28;240(1):27–39. doi: 10.1530/JOE-18-0479 (PMC6347282; doi:10.1530/JOE-18-0479)
Supplement: Supporting Table 1 [file JOE-18-0479-t001.pdf]

Supplementary Table 1

	Skeletal muscle and adipose		Liver		Brain
	Lean group	Obese group	Lean group	Obese and type 2 diabetes group	
Number	8 males	10 males	7 males	10 males	8 males
Age (years)	50.5 ± 3.3	50.0 ± 3.3	47.5 ± 6.0	52.3 ± 2.9	38.1 ± 5.8
Body mass index (kg/m^2)	23.7 ± 0.4	32.9 ± 0.9	23.5 ± 1.1	35.0 ± 1.0	24.9 ± 1.3
Arterialized cortisol-binding activity (nM)	281.5 ± 24.6	304.4 ± 50.6	398.4 ± 72.9	379.8 ± 94.4	296.5 ± 23.0
Arterialized 12G2 ELISA (nM)	277.0 ± 18.2	296.9 ± 44.2	400.8 ± 54.9	388.9 ± 81.9	282.0 ± 23.0
Arterialized 9G12 ELISA (nM)	250.0 ± 70.8	226.4 ± 67.1	338.8 ± 52.2	334.5 ± 29.1	262.1 ± 55.9
Arterial Blood Flow (L/min)	Muscle: 0.0027 ± 0.0013 Adipose: 0.0017 ± 0.0004	Muscle: 0.0034 ± 0.0013 Adipose: 0.0016 ± 0.0015	0.96 ± 0.17	1.22 ± 0.67	0.48 ± 0.23
Net uptake/release of cortisol binding capacity (nmol/min)*	Muscle: -0.014 ± 0.165 Adipose: -0.003 ± 0.077	Muscle: -0.148 ± 0.174 Adipose: 0.037 ± 0.117	15.6 ± 49.8	-46.8 ± 52.8‡	5.77 ± 10.36
Net uptake/release of 12G2 ELISA (nmol/min)*	Muscle: -0.015 ± 0.186 Adipose: 0.002 ± 0.084	Muscle: -0.083 ± 0.146 Adipose: 0.077 ± 0.159	44.0 ± 70.2	-56.6 ± 100.1	-7.60 ± 13.97
Net uptake/release of 9G12 ELISA (nmol/min)*	Muscle: -0.0003 ± 0.146 Adipose: -0.037 ± 0.110	Muscle: -0.079 ± 0.114 Adipose: -0.001 ± 0.040	10.3 ± 50.3	-29.7 ± 47.6	7.02 ± 18.16

Data are mean ± SD

‡p<0.005 by Wilcoxon signed rank test versus zero and p<0.001 by Mann Whitney U test versus the healthy group

* Net uptake/release = [arterial CBG – venous CBG (nmol/L)] x blood flow (L/min)
