# Supplementary material for: Neutrophil elastase-cleaved corticosteroid-binding globulin is absent in human plasma
Source: J Endocrinol. 2018 Sep 28;240(1):27–39. doi: 10.1530/JOE-18-0479 (PMC6347282; doi:10.1530/JOE-18-0479)
Supplement: Supporting Table 2 [file JOE-18-0479-t002.pdf]

Supplementary Table 2

Category	n	Age		Sex (n)	
		Mean(sd)	Range	Male	Female
Cardiovascular	21	65.1 (17.7)	32-94	10	11
Gastroenterological	7	70.25 (16.3)	45-85	1	6
Neurological	8	52.1 (23.8)	19-79	7	1
Respiratory	56	66.3 (15.7)	30-92	34	22
Sepsis	26	65.8 (17.5)	24-90	20	6
Surgical abdominal	10	71.7 (6.7)	60-82	5	5
Trauma	14	34.2 (21.1)	17-85	13	1
Other*	4	62.25 (9.0)	49-68	1	3

* Includes: foreign object/trauma/ENT, cardiothoracic surgical, malignancy
